# Sodium Butyrate (NaB) and Sodium Propionate (NaP) Reduce Cyclin A2 Expression, Inducing Cell Cycle Arrest and Proliferation Inhibition of Different Breast Cancer Subtypes, Leading to Apoptosis

**DOI:** 10.3390/biomedicines12081779

**Published:** 2024-08-06

**Authors:** José-Noel Ibrahim, Sandy El-Hakim, Josiane Semaan, Stéphanie Ghosn, Hiba El Ayoubi, Arpiné Ardzivian Elnar, Najat Tohme, Charbel El Boustany

**Affiliations:** 1 Department of Natural Sciences, School of Arts and Sciences, Lebanese American University (LAU), Beirut 1102, Lebanon; 2 College of Engineering and Technology, American University of the Middle East, Egaila 54200, Kuwait; sandy.el-hakim@aum.edu.kw; 3 Department of Laboratory Science, Faculty of Public Health—Branch 2, Lebanese University, Fanar 2611, Lebanon; josiane.semaan@hotmail.com (J.S.); stephanie.ghoson@gmail.com (S.G.); ayoubihiba@hotmail.com (H.E.A.); arpine.elnar@hotmail.fr (A.A.E.); ntohhar@ul.edu.lb (N.T.); celboustany@hotmail.com (C.E.B.)

**Keywords:** breast cancer, sodium propionate, sodium butyrate, cell cycle, apoptosis

## Abstract

Sodium butyrate (NaB) and sodium propionate (NaP) have recently garnered attention for their role in regulating inflammation and controlling signaling pathways of cell growth and apoptosis, potentially preventing cancer development. However, their therapeutic effect and the underlying mechanisms involved remain elusive in breast cancer. This study aims at investigating the anticancer role of NaB and NaP in different types of breast cancer by assessing their antiproliferative effect on MCF-7 and MDA-MB-231 cells (through an MTT assay), as well as their ability to alter the cell cycle and cyclin expression (using flow cytometry and RT-qPCR, respectively), and to promote apoptosis (using Annexin V-FITC conjugated and sub-G1 phase techniques). MDA-MB-231 cell proliferation was inhibited by NaB and NaP in a dose- and time-dependent manner with respective IC_50_ values of 2.56 mM and 6.49 mM. Treatment induced cell arrest in the G1 phase which was further supported by the significant reduction in cyclin A2 and cyclin B1 expressions. Finally, NaB, and less significantly NaP, induced apoptosis in a dose-dependent manner with higher concentrations required for MDA-MB-231 than MCF-7. Our findings elucidate the cyclin-dependent inhibitory effect of NaB and NaP on the progression of different breast cancer subtypes, thus highlighting their therapeutic potential in breast cancer.

## 1. Introduction

Breast cancer is responsible for over 2.3 million new cancer cases and 685,000 deaths of women annually worldwide [1]. There are five main intrinsic human breast cancer subtypes; these subtypes are mostly based on the genes that are differently expressed between them [2,3]. The first and most common subtype is luminal breast cancer. It is hormone receptor-positive (estrogen receptor- and/or progesterone receptor-positive), but HER2 (human epidermal growth factor receptor 2)-negative. This subtype has low-to-high levels of the protein Ki-67, which helps control the growth rate of cancer cells [2,3]. Moreover, it is typically a low-grade cancer that tends to grow slowly and has the best prognosis. The most commonly used cell line model for this subtype is MCF-7 [2,3]. Conversely, the triple-negative/basal-like subtype is an aggressive form of breast cancer that is hormone receptor-negative and HER2-negative; the MDA-MB-231 cell line is a good representative of this subtype [2,3].

Despite the considerable therapeutic success witnessed with chemotherapy, hormone therapy, and targeted therapy, breast cancer treatment remains challenged by drug resistance and the adversity of side effects, underscoring the need for safer and more efficient novel therapeutics. Short-chain fatty acids (SCFAs), produced through the microbiota fermentation of dietary fibers and non-digestible carbohydrates, are considered key regulators of the host immune response to microbial colonization by preventing inflammation and controlling phagocytosis and main signaling pathways of cell growth and apoptosis, thereby shaping the composition, integrity, and functionality of the intestinal epithelial barrier [4,5,6]. Accordingly, their preventive role in different types of cancer [7,8,9,10], especially colon cancer [11,12,13], is well established; however, their implication in breast carcinogenesis is still elusive. Indeed, we previously tested the effect of two SCFAs, namely, sodium propionate (NaP) and sodium butyrate (NaB), on the luminal subtype [14]. Interestingly, our results revealed that MCF-7 cells treated with NaP or NaB were arrested in the G1 phase, leading to a reduction in cell proliferation rates in a dose-dependent manner, as well as cell apoptosis [14]. A similar effect of NaB treatment was observed in the prostate hormone-dependent cell line LNCaP [8], and it was linked to the decrease in the expression of cell cycle regulatory proteins, such as cyclins and cyclin-dependent kinases. Similarly, the inhibition of cell cycle progression in HT-29 human colonic adenocarcinoma cells by NaB was due to the modulation of cell cycle regulatory proteins such as cyclin D and p21 [13]. However, the role of NaP on these cancer hallmarks remains uncharted territory. Moreover, its effect, in comparison to NaB, on different breast cancer subtypes, particularly triple-negative/basal-like breast cancer, has not yet been investigated. Therefore, the aim of this study is to explore the effect of NaB and NaP on MDA-MB-231 cell proliferation, cycle, and apoptosis, as well as their effect on the expression of cyclins in both cell lines, MCF-7 and MDA-MB-231.

## 2. Materials and Methods

All culture media and reagents were purchased from Sigma-Aldrich*™* (Ibra Haddad, Beirut, Lebanon).

### 2.1. Culture of MDA-MB-231 Cells

The human breast cancer MDA-MB-231 cell line was kindly provided by Pr. Hasnaa Bouharoun-Tayoun (Lebanese University, Faculty of Public Health, Fanar). Cells were cultured as previously described for MCF-7 [14]. MCF-10A cells were cultured in DMEM-F12 supplemented with horse serum (5%), hydrocortisone (0.5 μg/mL), EGF (20 ng/mL), insulin (10 μg/mL), 100 U/mL of penicillin, and 100 μg/mL of streptomycin. Cells were either left untreated (control) or incubated with different concentrations of NaB or NaP for 24, 48, 72, and 96 h.

### 2.2. Cell Proliferation Assay

MDA-MB-231 proliferation was assessed through an MTT assay as previously described for MCF-7 [14]. Cells were treated with different NaB or NaP concentrations ranging between 0.5 mM and 10 mM for 3 days, and proliferation rates were determined daily.

### 2.3. Cell Cycle Analysis

MDA-MB-231 distribution in G1-S-G2/M phase, after the addition of NaB or NaP for 48 h, was analyzed as previously described for MCF-7 [14].

### 2.4. Assessment of Apoptosis

Apoptosis assessment in MDA-MB-231 cells was performed using two different techniques. First, apoptosis was detected by staining living cells with both cell permeant Hoechst 33342 and Annexin V-FITC conjugates. Following treatment with NaB or NaP for 48 h, living cells were stained in the dark with Hoechst for 15 min at 37 °C in an atmosphere containing 5% CO_2_ [15,16]; this dye will stain all cells. The cells were then washed twice with phosphate-buffered saline (PBS), and Annexin V-FITC conjugate was added for 15 min, followed by another wash with PBS to remove the surplus. Apoptotic cells that were positive for Annexin V-FITC conjugate versus unstained non-apoptotic cells were counted on an epi-fluorescent microscope. Second, apoptosis was assessed by cell cycle sub-G1 phase as previously described for MCF-7 [14].

### 2.5. Real-Time PCR

MCF-7 and MDA-MB-231 cell lines were cultured for 24 h and then grown for 72 h with 1 mM NaB or 5 mM NaP, before RNA extraction in 6-well plates containing DMEM. After washing twice with 1 mL of PBS, cells were centrifuged and the supernatant was discarded. An appropriate volume of TRI reagent was added to cells (8 to 10 × 10^4^ cells) based on the manufacturer’s protocol (Sigma Aldrich, St. Louis, MO, USA). At the end of the extraction procedure, the RNA pellet was dissolved in 20 µL of RNase free water. RNA purity, quality, and quantity were measured by Nanodrop 1000 Spectrophotometer (Thermo Scientific, Wilmington, DE, USA) at 260 and 280 nm. Primer sequences were selected using the Primer-BLAST Genbank (https://www.ncbi.nlm.nih.gov/tools/primer-blast/ accessed on 25 July 2024). Eight target genes were quantified; cyclins D1, D2, E1, E2, A1, A2, B1, and B2 expression levels were compared to those of the three housekeeping genes β-actin (*ACTB*), peptidylprolyl isomerase A (*PPIA*), and ubiquitin C (*UBC*). One µg of total RNA was reverse-transcribed in a final volume of 20 µL using ReadyScript™ cDNA Synthesis Mix (final concentration 1X; Sigma Aldrich) containing optimized concentrations of MgCl_2_, dNTPs, recombinant RNase inhibitor protein, ReadyScript reverse transcriptase, random primers, oligo(dT) primer, and stabilizers. Amplification was performed under standard conditions (5 min at 25 °C, 30 min at 42 °C, and 5 min at 85 °C). Real-time PCR was performed using SYBR Green quantitative RT-PCR kit (Sigma Aldrich). PCR amplifications were performed at 95 °C for 5 min (1 cycle), followed by 45 cycles of incubation at 95 °C for 10 s and 56–57 °C for 10 s in the LightCycler 96 machine (La Roche, Basel, Switzerland). Primer specificity was confirmed by means of agarose gel electrophoresis. The housekeeping gene used as an internal reference was β-actin for both cell lines, selected using the geNorm software, version 3.5 [17]. The relative expression was calculated using the 2^−ΔΔCt^ method.

### 2.6. Statistical Analyses

Statistical analyses were performed using the Microcal Origin software, version 7.0 (Microcal Software Inc., Northampton, MA, USA). All data followed a Gaussian distribution and were presented as mean ± SEM (standard error of the mean). Student’s *t*-test was used to compare differences between treated and non-treated MDA-MB-231 or MCF-7 cells. *p* values less than 0.05 were considered significant. All experiments were repeated at least three times.

## 3. Results

### 3.1. MDA-MB-231 Cell Proliferation Was Reduced by NaB and NaP Treatment in a Dose- and Time-Dependent Manner

Prior to MDA-MB-231 proliferation assessment, the cell toxicity of NaB and NaP was tested using a trypan blue exclusion assay. No cytotoxicity was observed in MCF-7 and MDA-MB-231 cells which is in line with the findings of Salimi et al. who previously reported no cytotoxicity of NaB, even at 10 mM and 20 mM, in MCF-7 and MDA-MB-468 cell lines [18].

**Figure 1:**
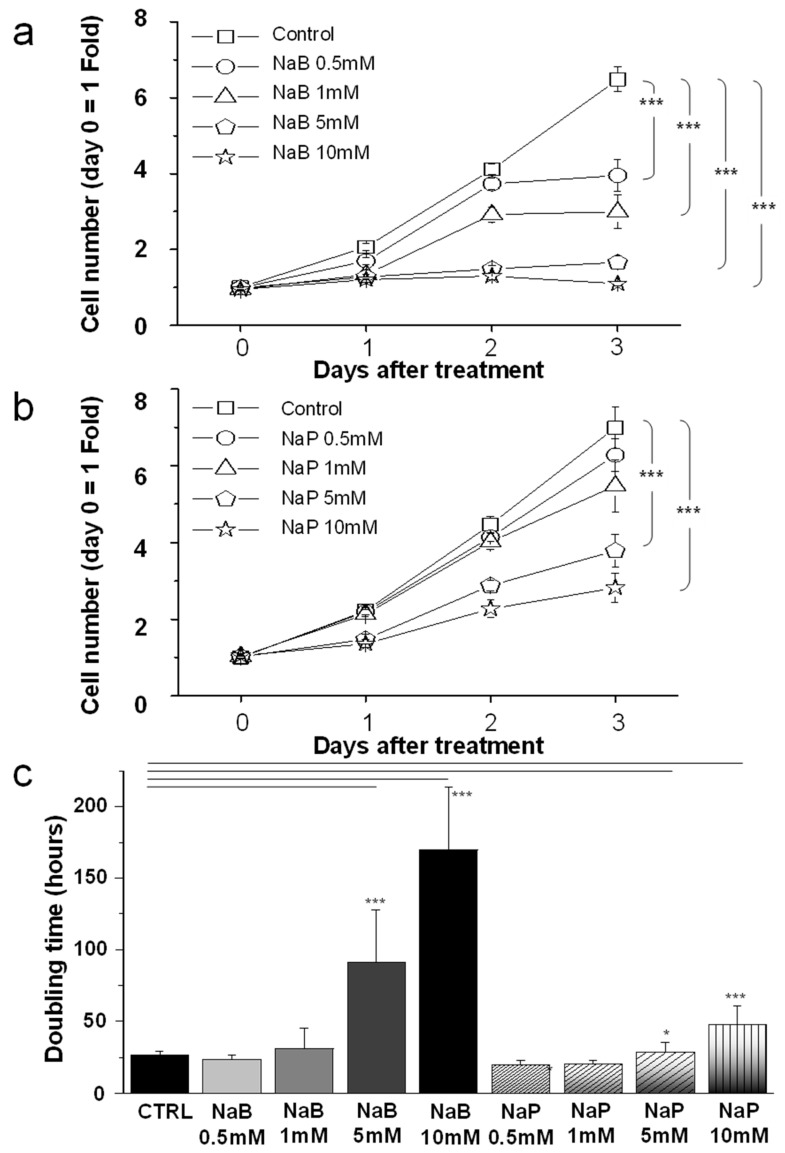
Inhibitory effect of NaB and NaP on cell proliferation. Cell number was determined from OD absorbance in MDA-MB-231 cells treated with different concentrations of (**a**) NaB (0.5, 1, 5, or 10 mM) or (**b**) NaP (0.5, 1, 5, or 10 mM). Absorbance was measured daily for 3 days after treatment. (**c**) Cell doubling time after treatment of MDA-MB-231 with different concentrations of NaB or NaP. Data are obtained from three different experiments and are represented as mean ± SEM. *p* values were calculated using Student’s *t*-test. * *p*< 0.05; *** *p*< 0.001.

Cells treated with different concentrations of NaB or NaP presented a dose- and time-dependent decrease in proliferation. Indeed, after 3 days of NaB treatment, cells showed a 39% decrease in proliferation at 0.5 mM, 53.8% at 1 mM, 74.5% at 5 mM, and 83.1% at 10 mM (Figure 1a). Similarly, treatment with NaP resulted in a 10.3% decrease in MDA-MB-231 cell proliferation at 0.5 mM, 21.7% at 1 mM, 45.6% at 5 mM, and 59.6% at 10 mM (Figure 1b). Based on these results, we generated the doubling time of the population after treatment with different concentrations of NaB or NaP (Figure 1c). Control cells showed a population doubling time of 27 h, whereas treatment with 5 mM of NaB and 10 mM of NaP remarkably increased the doubling time to 91 h and 47 h, respectively. We also calculated the half-maximal inhibitory concentration (IC_50_) of NaB and NaP on MDA-MB-231 cells (Supplementary Figure S1). Using the Origin Lab software, version 7.0 (Originlab Corporation, Northampton, MA, USA), a logarithmic fitting of the curves was performed based on the following mathematical formula: y = A1 + (A2 − A1)/(1 + 10^((LOG(x0) − x)×p)^), where IC_50_ is equal to LOG(x_0_). Results showed that NaB had an IC_50_ of 2.56 mM, whereas NaP had an IC_50_ of 6.49 mM, hence indicating a more potent inhibitory effect by NaB on MDA-MB-231 cells. Moreover, treatment with NaB or NaP for 72 h displayed changes in MDA-MB-231 morphology similarly to what was previously shown in MCF-7 (Supplementary Figure S2) [14]. Namely, MDA-MB-231 cell treatment with either compound led to a post-mitotic neuron-like differentiation [19,20]. Interestingly, NaB and NaP had a weaker effect on the normal human breast cell line MCF-10A proliferation. Indeed, their inhibitory effect was mainly noted when cells were treated for 72 h with very high concentrations of NaB (20 mM) or NaP (30 mM). Accordingly, the calculation of the IC50 of NaB and NaP revealed estimated values of 76.6 mM and 83.3 mM, respectively, which are 30 and 13 times higher than those observed in MDA-MB-231 cells, hence indicating the greater selectivity of these compounds for breast cancer cells (Supplementary Figure S3).

### 3.2. NaB and NaP Treatment Induced Cell Cycle Arrest and Blockage of MDA-MB-231 Cells in G1 Phase

**Figure 2:**
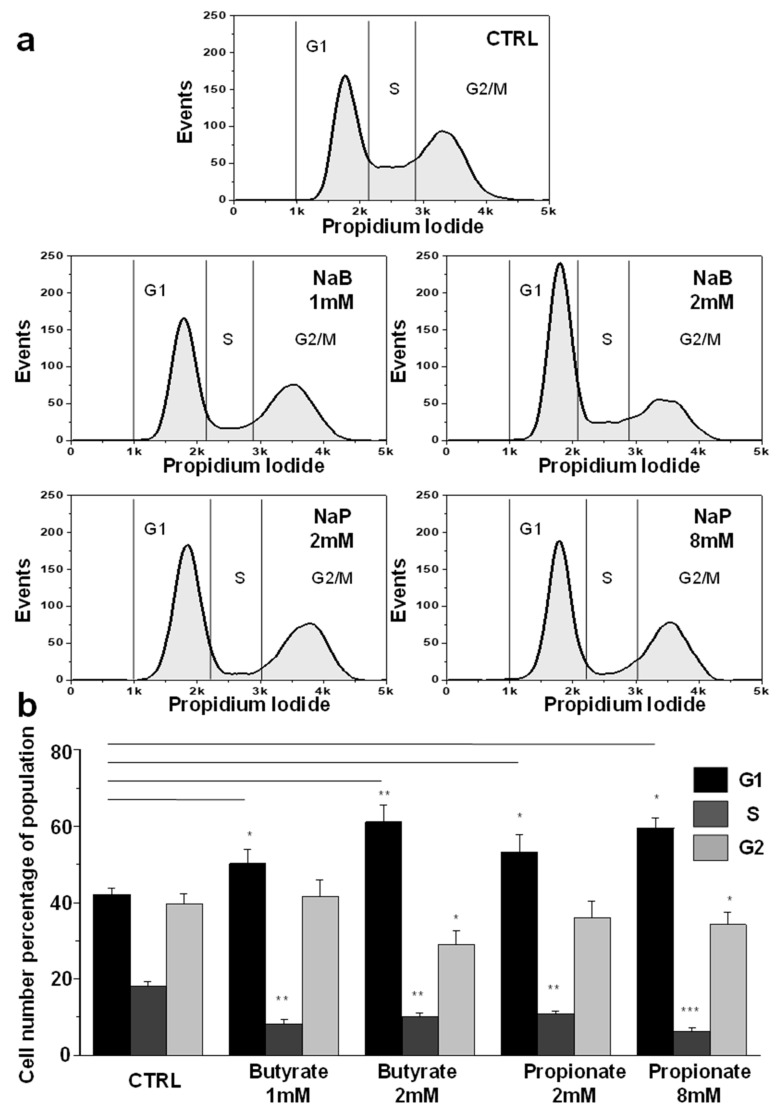
Cell cycle analysis of MDA-MB-231 cells treated with NaB or NaP. (**a**) The upper panel shows the control cell cycle profile in MDA-MB-231 cells. The middle panels display the effect of 1 mM (left panel) and 2 mM (right panel) NaB on cell cycle shape. The lower panels show the effect of 2 mM (left panel) and 8 mM (right panel) NaP on cell cycle profile. (**b**) Analysis of cell cycle using flow cytometry in controls and cells treated with different concentrations of NaB or NaP for 48 h. Histogram summarizing the distribution of the cells in G_1_, S and, G2/M phases. Data are obtained from three different experiments and are represented as mean ± SEM. *p* values are calculated using Student’s *t*-test. * *p* < 0.05; ** *p* < 0.01; *** *p* < 0.001.

The effect of NaB and NaP on the MDA-MB-231 cell cycle was further studied. Treatment with different concentrations of NaB (1 and 2 mM) or NaP (2 and 8 mM) for 48 h resulted in a blockage of MDA-MB-231 cells in the G1 phase of the cell cycle (Figure 2a). The proportion of cells in the G1 phase treated with 1 or 2 mM of NaB increased significantly by 19.5% and 44.8%, respectively, whereas the cell number in the S phase decreased by 55% in both concentrations. Similarly, the number of cells in the G2/M phase was significantly reduced by 27% in the group treated with 2 mM of NaB compared to the untreated group (Figure 2b). Likewise, a significant increase in the percentage of cells in the G1 phase (26.3% and 41%), and a decrease in the number of cells in the S phase (40.8% and 65.3%) were observed after treatment with 2 or 8 mM of NaP compared to the control group. In addition, the number of cells in the G2/M phase was 13.8% lower in the group treated with 8 mM of NaP compared with the untreated group (Figure 2b). Our findings suggest that both NaB and NaP induce cell cycle arrest in G1 in a dose-dependent fashion.

### 3.3. NaB and NaP Treatment Induced Cell Cycle Arrest in MCF-7 and MDA-MB-231 by Reducing Cyclin A2 Gene Expression

**Figure 3:**
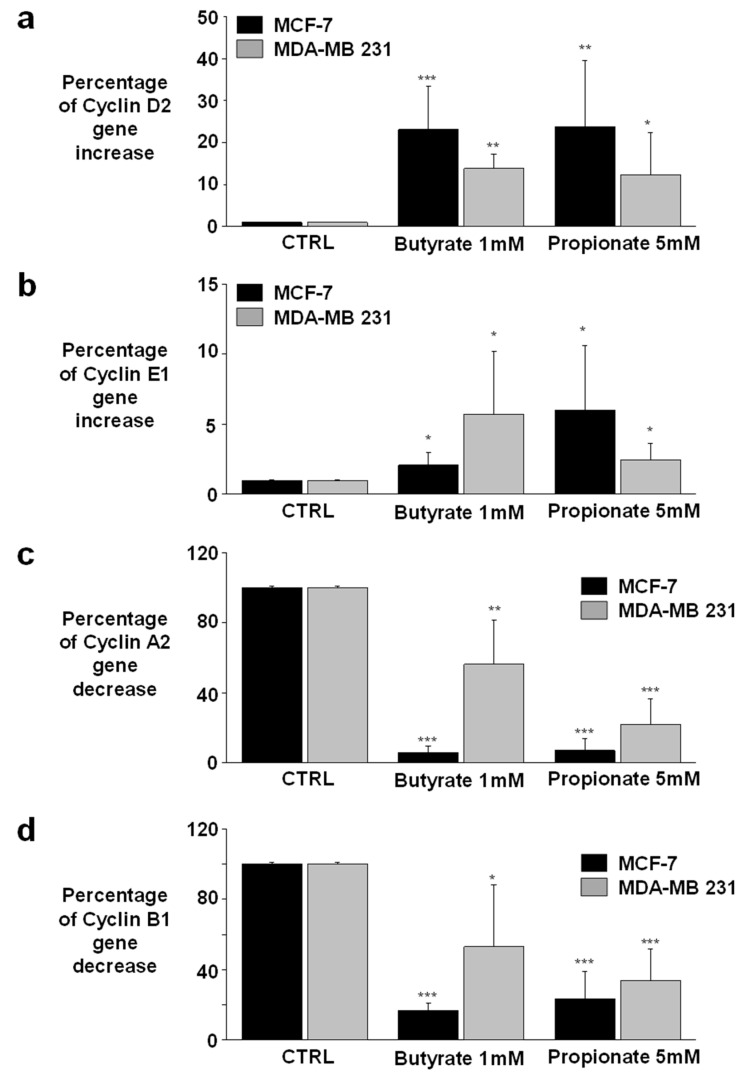
Effect of NaB and NaP on cyclin gene (D2, E1, A2, and B1) expression in MDA-MB-231 and MCF-7 cell lines. Expression levels were analyzed using quantitative real-time PCR in MCF-7 and MDA-MB-231 untreated cells and those treated for 72 h with 1 mM NaB or 5 mM NaP. The histograms represent the percentage of gene increase in (**a**) cyclin D2 and (**b**) cyclin E1 and the percentage of gene decrease in (**c**) cyclin A2 and (**d**) cyclin B1 in comparison to controls. Values are represented as mean ± SEM and analyzed using Student’s *t*-test. * *p* < 0.05; ** *p* < 0.01; *** *p* < 0.001.

Cyclins are the core regulation system of the cell cycle; thus, any changes in their expression would have direct consequences on the cell cycle. In this context, the expressions of different cyclins (D1, D2, E1, E2, A1, A2, B1, and B2) were evaluated in the present study. Cyclins D1, E2, A1, and B2 were not expressed in both cell lines, whereas cyclins D2, E1, A2, and B1 were present in both MCF-7 and MDA-MB-231 cells (Figure 3). Therefore, we studied their expression before and after treatment with 1 mM NaB or 5 mM NaP. Interestingly, the treatment induced a significant increase in cyclin D2 expression compared to the control (NaB: 23.1% ± 10.2 and 13.9% ± 3.3, respectively; NaP: 23.7% ± 15.9 and 12.4% ± 10, respectively) (Figure 3a). The expression of cyclin E1 was also increased in MCF-7 and MDA-MB-231 cells treated with NaB (2.1% ± 0.9 and 5.7% ± 4.5, respectively) or NaP (6.0% ± 4.6 and 2.5% ± 1.2, respectively) compared to the control (Figure 3b). In contrast, the expression levels of cyclin A2 were significantly decreased in MCF-7 and MDA-MB-231 cells treated with NaB (6.0% ± 3.7 and 56.4% ± 25.4, respectively) or NaP (7.2% ± 6.6 and 22% ± 14.6, respectively) as compared to the untreated group (Figure 3c). Finally, the treatment of MCF-7 and MDA-MB-231 with NaB induced a significant decrease in cyclin B1 expression with respective levels of 17% ± 4.1 and 53.1% ± 35.2 compared to the controls. Similarly, NaP significantly reduced cyclin B1 expression levels in MCF-7 (23.4% ± 15.6) and MDA-MB-231 (34% ± 17.8) in comparison to untreated cells (Figure 3d). Taken together, these results indicate that cell cycle arrest occurs at the end of the G1 phase, directly before the beginning of the S phase. In addition, this comparative study of cyclins showed that luminal breast cancer is more sensitive to treatment with SCFAs than the triple-negative/basal-like subtype.

### 3.4. NaB and NaP Treatment Induced Cell Apoptosis in MDA-MB-231 Cells

**Figure 4:**
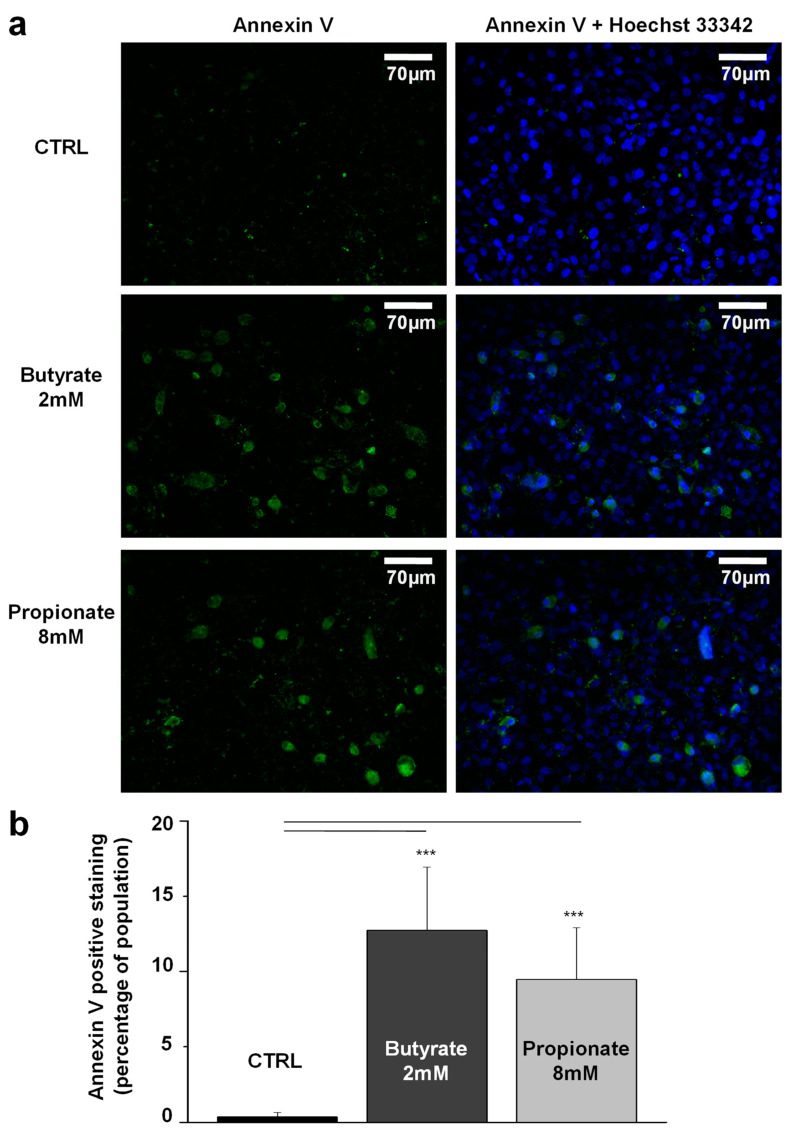
Effect of medium levels of NaB (2 mM) or NaP (8 mM) on MDA-MB-231 cell apoptosis assessed by means of Annexin V-FITC and Hoechst staining. (**a**) The upper panel shows the stained nuclei or plasma membrane of MDA-MB-231 control cells. The middle panel shows the effect of 2 mM NaB and 8 mM NaP (lower panel) after 48 h of cell treatment. (**b**) Histogram summarizing the percentage of Annexin V-FITC-positive cells in controls versus cells treated with 2 mM NaB or 8 mM NaP for 48 h. Data are obtained from three different experiments and are represented as mean ± SEM. *p* values are analyzed using Student’s *t*-test. *** *p* < 0.001.

**Figure 5:**
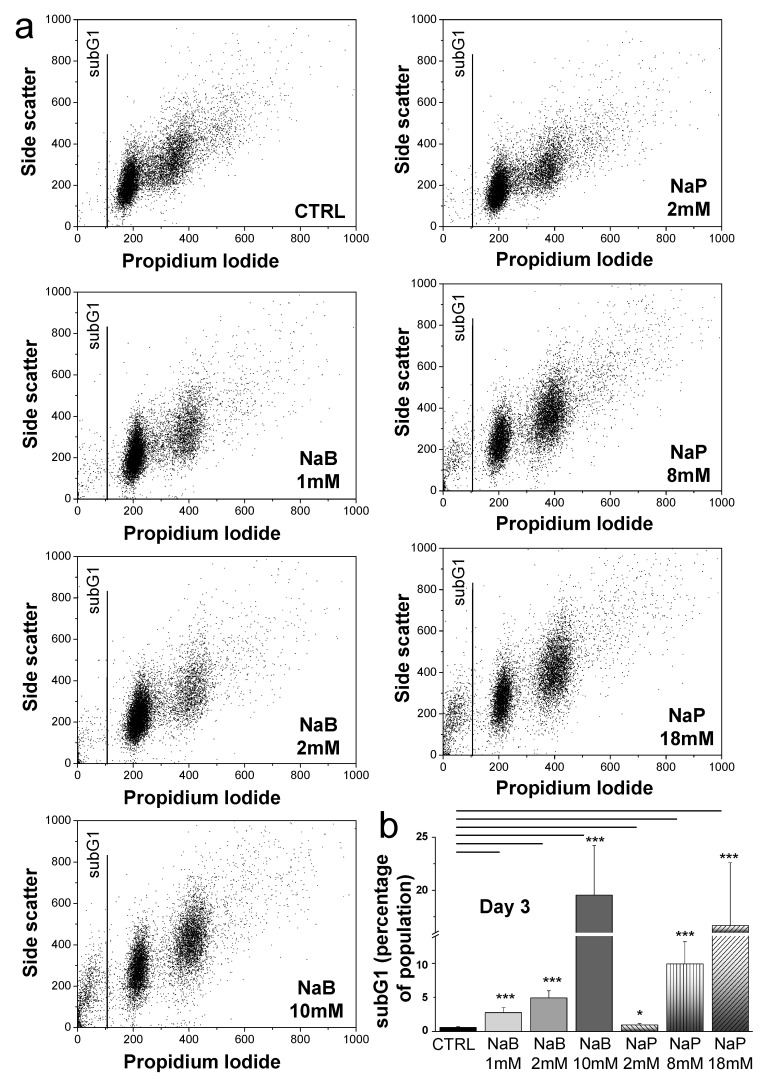
Effect of NaB and NaP on MDA-MB-231 cell apoptosis. (**a**) The panels show the FACS analysis of untreated MDA-MB-231 cells in the sub-G1 phase versus those treated for 72 h with different concentrations of NaB (1, 2, and 10 mM) or NaP (2, 8, and 18 mM). (**b**) Histogram summarizing the percentage of untreated and 72 h treated MDA-MB-231 cells in the sub-G1 phase. Data are obtained from three different experiments and are represented as mean ± SEM. *p* values are calculated using Student’s *t*-test. * *p* < 0.05; *** *p* < 0.001.

To evaluate the apoptotic effect of NaB and NaP in MDA-MB-231, treated cells were analyzed either using Annexin V-FITC conjugated to detect the (phosphatidylserine) PS translocation at the plasma membrane or using sub-G1 phase analysis through flow cytometry. The percentage of Annexin V-FITC positive cells significantly increased from 0% in the control group to 13% and 6% after 48 h treatment with NaB (2 mM) and NaP (8 mM), respectively (Figure 4), hence indicating that treatment with concentrations close to the IC_50_ induces early apoptosis in the MDA-MB-231 cell line. Moreover, the analysis of the cell cycle following treatment with NaB (1 and 2 mM) or NaP (2 and 8 mM) revealed the appearance of a sub-G1 population that increases in a dose-dependent manner (Figure 5a). This population presents altered DNA content generally associated with cells undergoing late apoptosis. Indeed, our results showed that a 72-h treatment with low doses of NaB (1 mM) induced low apoptosis (4%), whereas treatment with a low dose of NaP (2 mM) was unable to induce a significant amount of cell death. However, the rate of apoptosis significantly increased in cells treated with 2 mM or 10 mM of NaB (5% and 20%, respectively), and 8 mM or 18 mM of NaP (15% and 18%, respectively) (Figure 5b). Overall, our data indicate that both NaB and NaP can induce apoptosis in the MDA-MB-231 cell line in a dose-dependent fashion.

## 4. Discussion

In recent years, studies have found that the gut microbiota composition is significantly altered in cancer patients compared to that in healthy people [21] and showed that metabolites derived from the gut microbiota, in particular SCFAs, inhibit tumor growth by preventing inflammation, regulating immune response, and controlling cancer-specific signaling pathways [5,22]. Moreover, SCFAs were found to effectively suppress cancer cell growth and metastasis via the inhibition of histone deacetylase (HDAC) activity and the regulation of oncogene/tumor-suppressor gene expression [23,24,25,26]. Accordingly, histone deacetylase inhibitors (HDACi) have gained considerable interest as novel and promising therapeutic agents, given their notable anti-cancer effects [23,24,26].

Indeed, numerous studies have demonstrated that SCFAs, by acting as HDACi, are able to inhibit cell proliferation, mainly in colorectal cancer, thus leading to cell cycle arrest and the induction of programmed cell death or apoptosis [11,13,26,27,28,29,30]. NaB was further shown to effectively inhibit the cell proliferation of human prostate cancer cells and induce apoptosis [8]. Similarly, it appears that NaB treatment in tongue cancer is able to suppress the *BIM* oncogene which is abnormally overexpressed, and its overexpression is associated with aggressive malignant features [31]. Finally, it has been reported that NaB regulates breast cancer growth and induces apoptosis by targeting different signaling pathways [18,27,32]. Similar to NaB, NaP has been shown to inhibit lung cancer cell proliferation by inducing cell cycle arrest, particularly in the G2/M phase by regulating survivin and p21 expression [33].

In the present study, we studied the effect of NaB and NaP on the proliferation of MDA-MB-231 cells and demonstrated that both molecules exhibit a significant dose- and time-dependent antiproliferative effect through the blockage of cells in the G1 phase of the cell cycle and the induction of apoptosis. NaB demonstrated greater potency than NaP, as shown by its lower IC_50_. This effect was observed in our previous work on the MCF-7 cell line [14]. The inhibitory effect of NaB was also reported by Rodrigues et al. (2015), who showed that MCF-7 and MDA-MB-231 are both sensitive to treatment with 2 mM NaB via a colony formation assay [34].

Prior research has shown that NaB stimulates cyclin D and p21 and inhibits cyclin-dependent kinase 2 expression in HT-29 colonic epithelial cells [13], and that HDAC inhibition leads to cell cycle arrest in G1 and G2 [25]. In this context, we investigated the effect of NaB and NaP treatment on the expression of cell cycle regulators, namely, cyclins, in MCF-7 and MDA-MB-231 cells. Only cyclins D2, E1, A2, and B1 were expressed in both cell lines; cyclins D and E1 were overexpressed following treatment, while cyclins A2 and B1 were underexpressed. This particular cyclin profile is in favor of a blockage of cells in the late G1 phase (early S phase). Indeed, the overexpression of cyclins D2 and E1 indicates that the cells can enter and complete the G1 phase [35,36]. However, the underexpression of A2 means that the progression through the S phase is impaired since the latter cyclin is essential for the completion of the S phase and the entry in the G2 phase [35,36]; hence, cells tend to accumulate in the early S phase. Accordingly, the number of cells in the G2 phase is decreased, thus reducing the expression of cyclin B1.

On the other hand, NaB and NaP treatment induced a change in the form of MDA-MB-231 cells, transforming them into a neuron-like phenotype; the same morphological change was also seen in our previously published work on MCF-7 [14]. In this case, cancer cells undergo a terminal differentiation that can be due to the inhibition of certain HDACs [37].

In terms of apoptosis, and in agreement with our previous findings on MCF-7 [14], our data revealed that NaB and NaP are also potent growth inhibitors of the hormone-independent MDA-MB-231 cells. There are multiple mechanisms associated with NaB-induced apoptosis in cancer. For instance, in breast cancer, NaB promotes apoptosis either through reactive oxygen species (ROS) formation and mitochondrial impairment [18], or via the activation of the Fas/FasL system in a P53-independent manner [32]. The apoptotic effect of NaB in colorectal carcinoma cells [19,38] is mediated by the mTOR/S6K1 pathway [38]. In other cancer cells, namely, chronic myeloid leukemia cells, NaB was found to induce apoptosis via the activation of caspase 8 and caspase 9, which are key mediators of the extrinsic and intrinsic apoptotic pathways, respectively [39]. Another study, conducted by Taylor and his colleagues (2019), demonstrated that NaB increased apoptosis in glioblastoma cells by decreasing antiapoptotic protein Bcl-2, increasing proapoptotic protein Bax, activating caspase 3, and degrading poly (ADP-ribose) polymerase (PARP) [40]. Similar to NaB, NaP was found to have an apoptotic effect on colon cancer via the regulation of PRMT (Protein arginine methyltransferases) [41]. Apoptosis was linked to caspase 8 activation, and it was controlled by survivin as well as p21 expression in lung cancer cells [33]. In accordance with these findings, Yoon and Ahn (2007) observed that the growth of Chinese hamster ovary cells was decreased in a dose-dependent manner in the presence of NaP by enhancing the production of follicle-stimulating hormone (FSH) [42].

## 5. Conclusions

Our results indicate that SCFAs could inhibit the progression of different breast cancer cells through the modulation of proliferative and cell cycle pathways, leading to cyclin A2- and B1-dependent cell arrest and apoptosis. Even though our research yielded valuable insights, we acknowledge that it presents some limitations. As our study was performed in vitro, these findings need to be replicated in ex vivo and in vivo models to better assess the effects and potential clinical applications of NaB and NaP. Moreover, as the mechanisms of action by which these SCFAs exert their antiproliferative and apoptotic effects are not fully elucidated, it is necessary to carry out further molecular investigations on the specific apoptotic pathways, markers, and proteins involved, as well as on epigenetic modulators. The signaling pathways underlying the inhibition of cyclins A2 and B1 and their regulators, such as p21, p27, and p53, by NaB and NaP should also be addressed. Finally, it would be interesting to study the anti-inflammatory effects of NaB and NaP and their potential role in regulating immune cell migration, adhesion, and cytokine secretion in breast cancer.

## Data Availability

The data presented in this study are available from the corresponding author on reasonable request.

## Supplementary Materials

The following supporting information can be downloaded at: https://www.mdpi.com/article/10.3390/biomedicines12081779/s1, Figure S1: The half maximal inhibitory concentration (IC_50_) of NaB and NaP in MDA-MB-231 cells; Figure S2: Effect of treatment with NaB or NaP on MDA-MB-231 cell morphology; Figure S3: The effect of NaB and NaP treatment on MCF-10A proliferation.
