# Supplementary material for: Sodium Butyrate (NaB) and Sodium Propionate (NaP) Reduce Cyclin A2 Expression, Inducing Cell Cycle Arrest and Proliferation Inhibition of Different Breast Cancer Subtypes, Leading to Apoptosis
Source: Biomedicines. 2024 Aug 6;12(8):1779. doi: 10.3390/biomedicines12081779 (PMC11351769; doi:10.3390/biomedicines12081779)
Supplement: Supplementary file 1 [file biomedicines-12-01779-s001.zip › biomedicines-3132732-supplementary.pptx]

## Slide 1
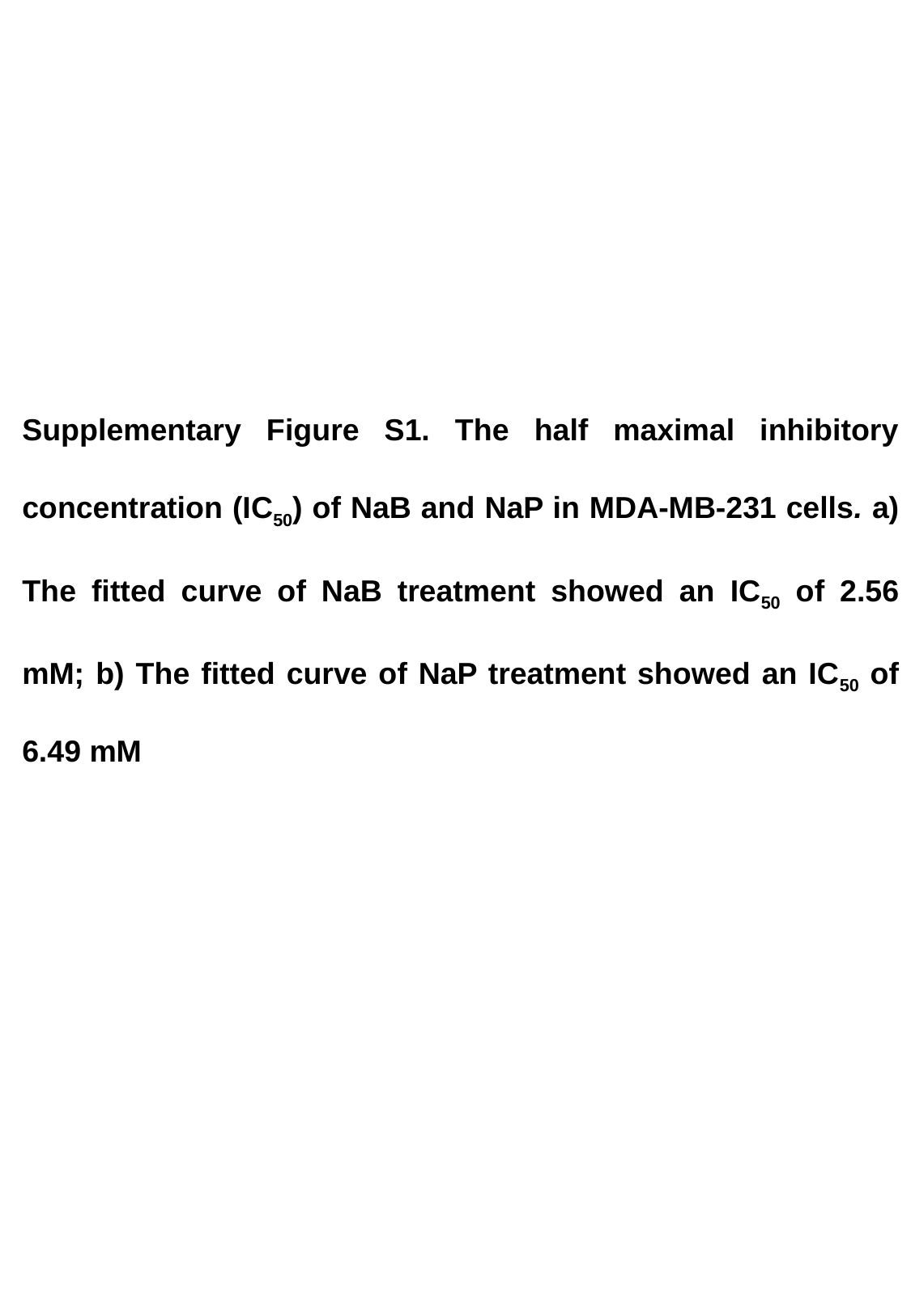

Supplementary Figure S1. The half maximal inhibitory concentration (IC50) of NaB and NaP in MDA-MB-231 cells. a) The fitted curve of NaB treatment showed an IC50 of 2.56 mM; b) The fitted curve of NaP treatment showed an IC50 of 6.49 mM

## Slide 2
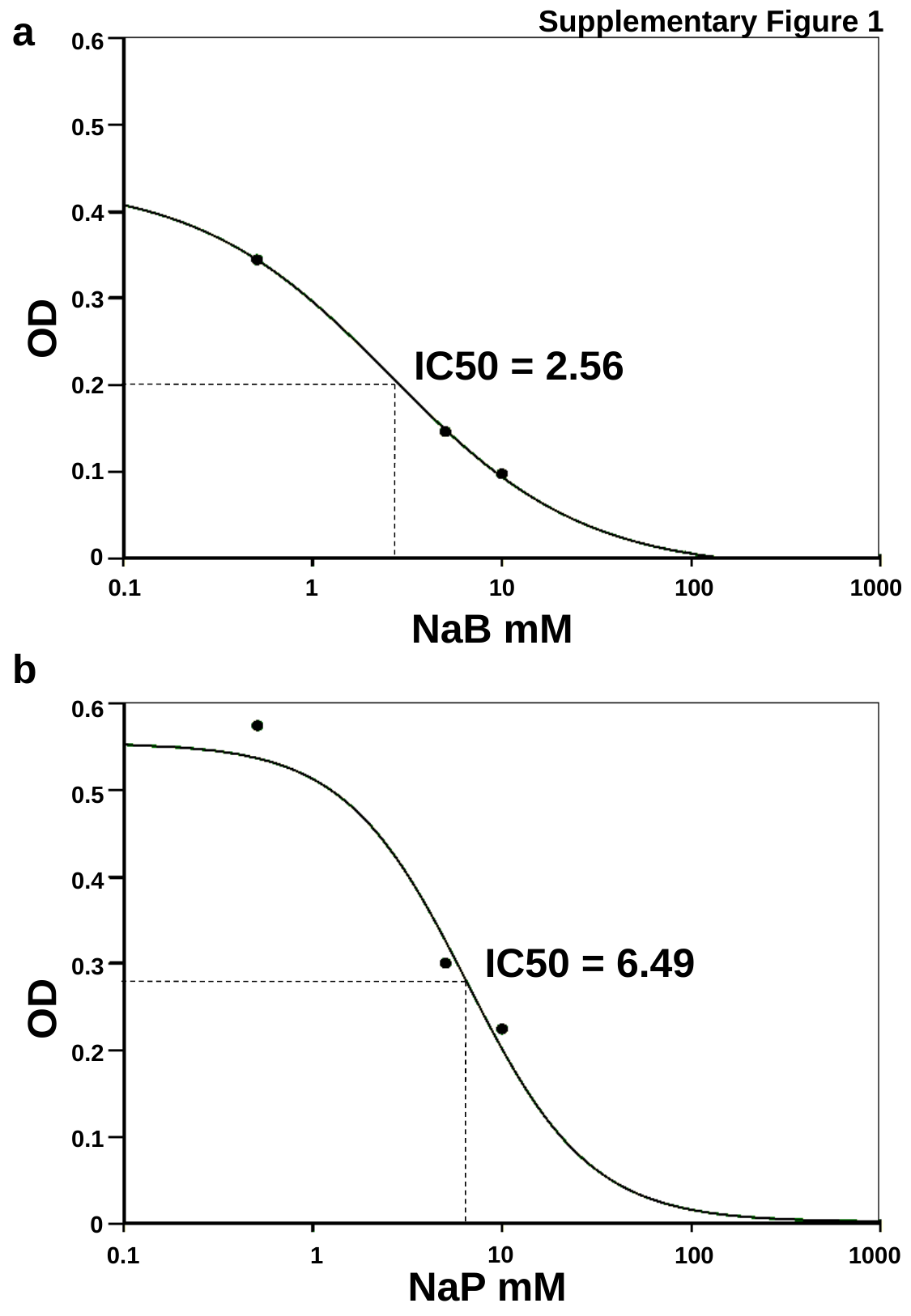

Supplementary Figure 1
a
0.6
0.5
0.4
0.3
OD
IC50 = 2.56
0.2
0.1
0
10
0.1
1
 100
1000
NaB mM
b
0.6
0.5
0.4
IC50 = 6.49
0.3
OD
0.2
0.1
0
10
0.1
 1
 100
1000
NaP mM

## Slide 3
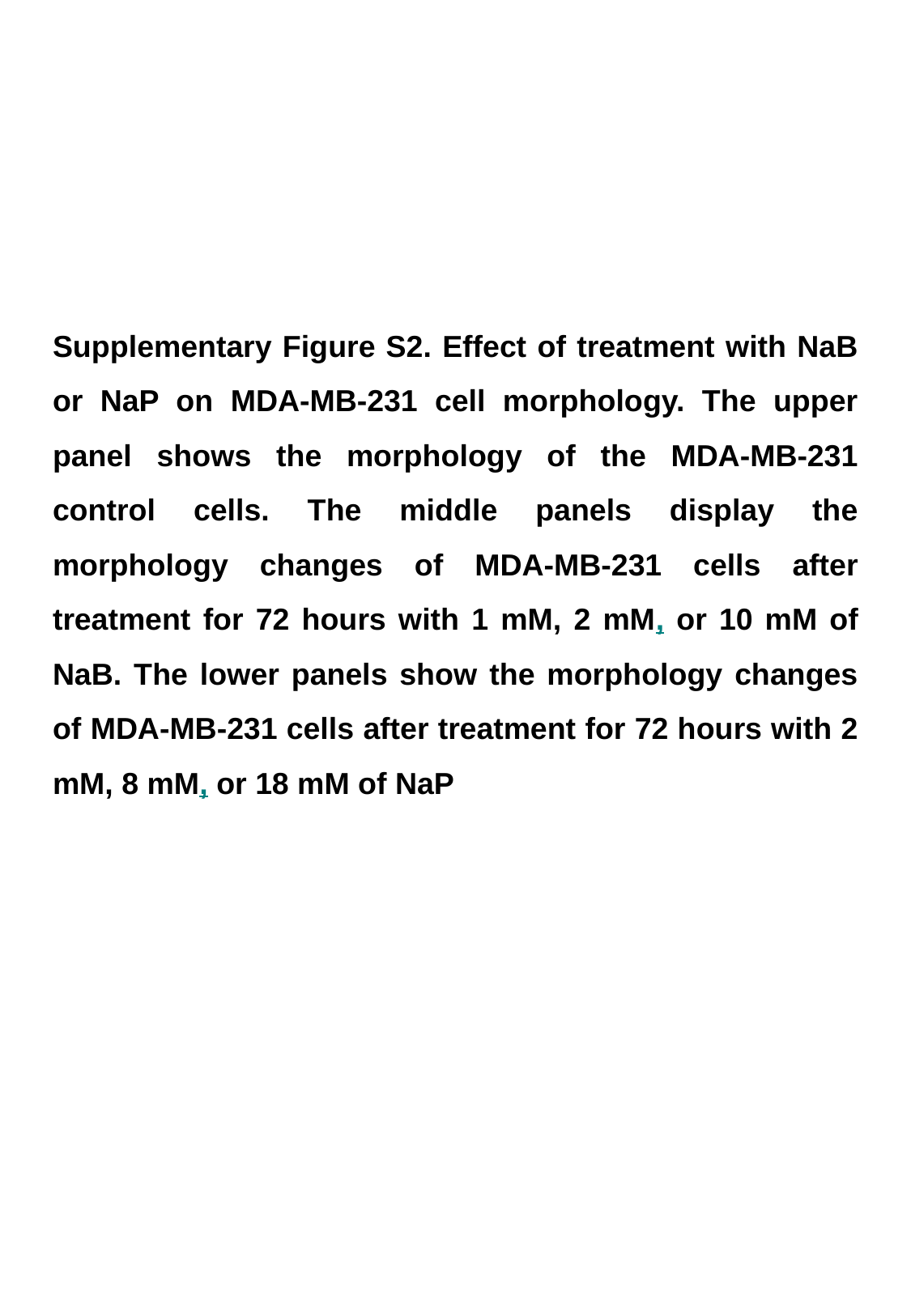

Supplementary Figure S2. Effect of treatment with NaB or NaP on MDA-MB-231 cell morphology. The upper panel shows the morphology of the MDA-MB-231 control cells. The middle panels display the morphology changes of MDA-MB-231 cells after treatment for 72 hours with 1 mM, 2 mM, or 10 mM of NaB. The lower panels show the morphology changes of MDA-MB-231 cells after treatment for 72 hours with 2 mM, 8 mM, or 18 mM of NaP

## Slide 4
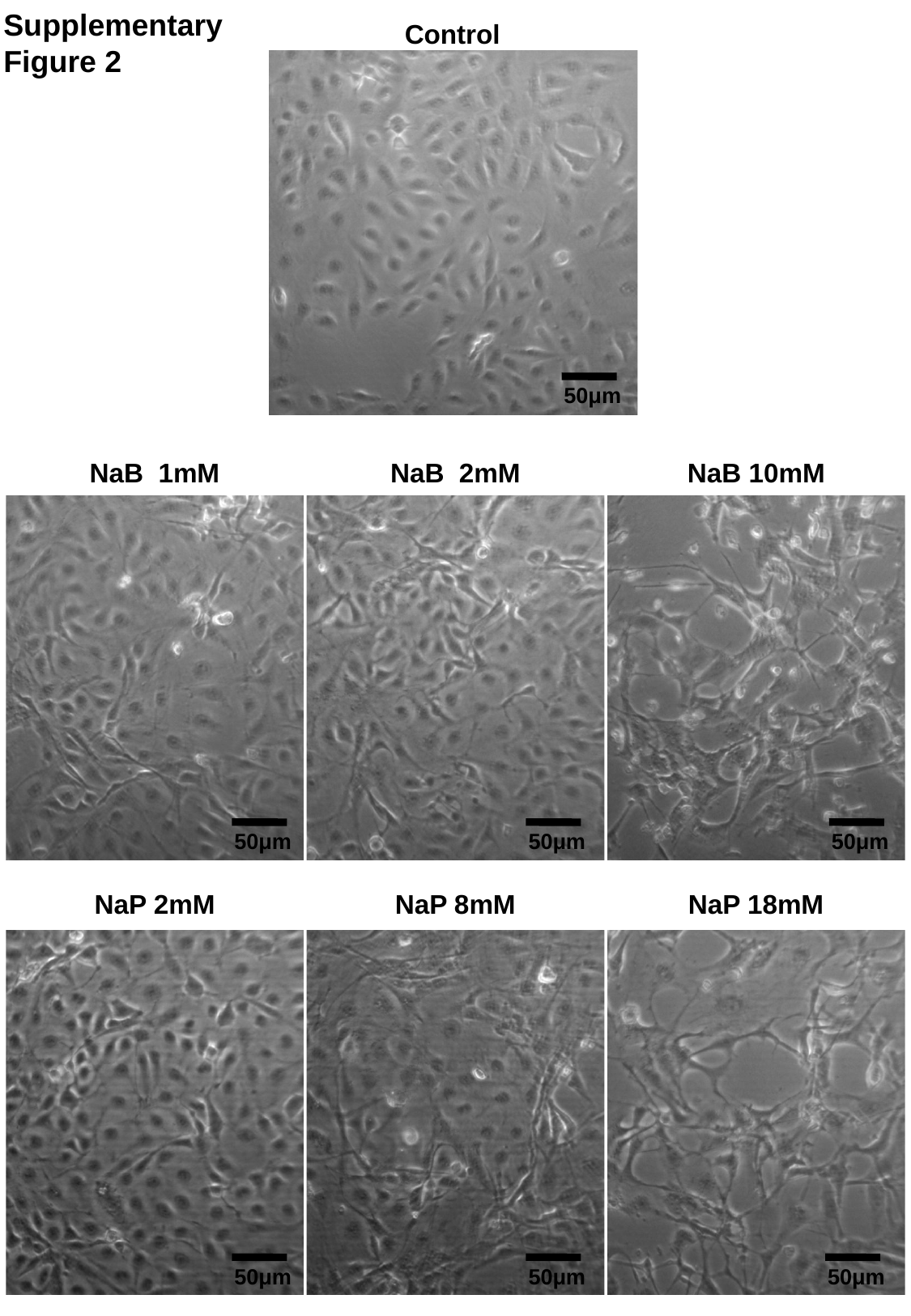

Supplementary Figure 2
Control
50μm
NaB 1mM
NaB 2mM
NaB 10mM
50μm
50μm
50μm
NaP 2mM
NaP 8mM
NaP 18mM
50μm
50μm
50μm

## Slide 5
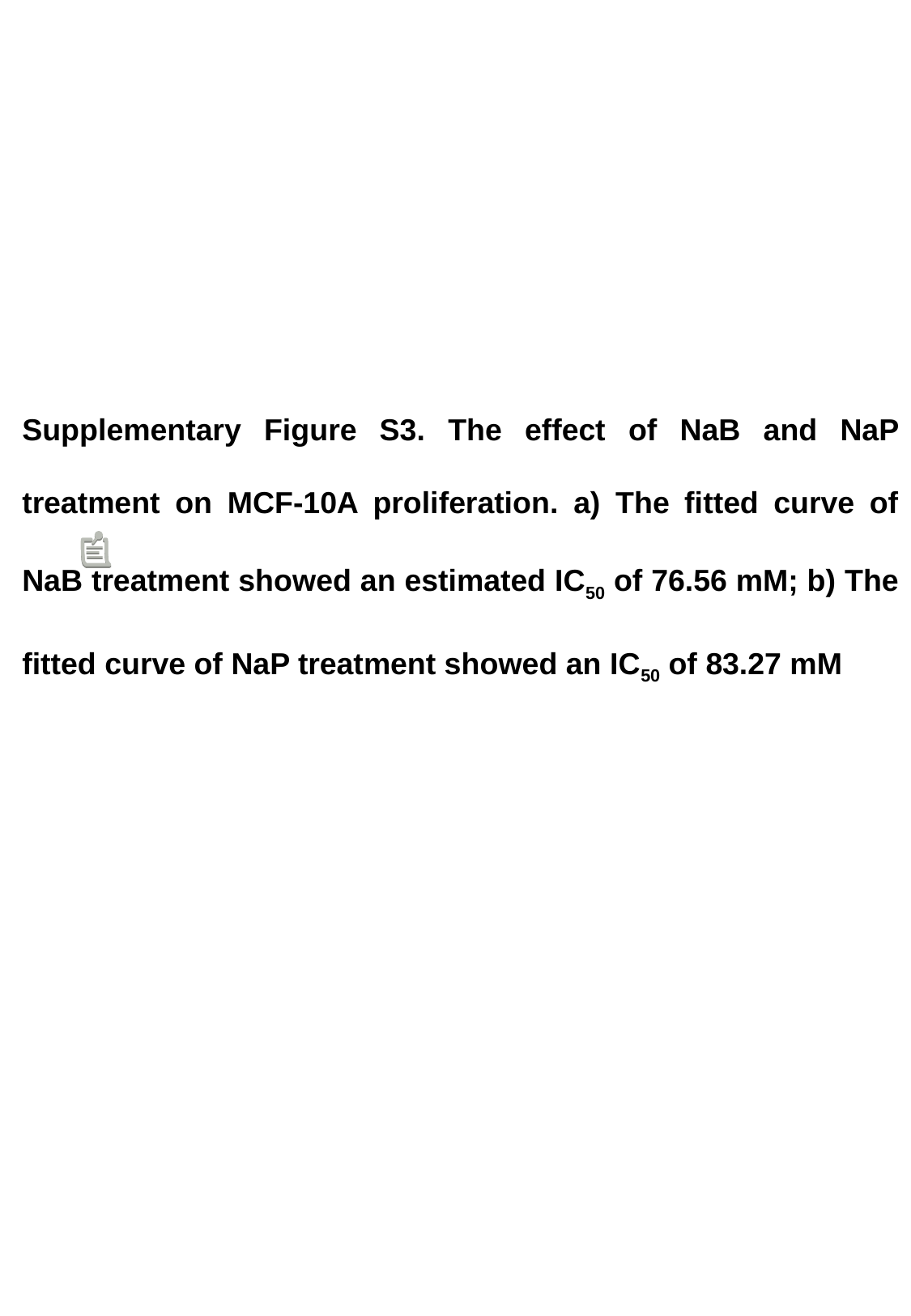

Supplementary Figure S3. The effect of NaB and NaP treatment on MCF-10A proliferation. a) The fitted curve of NaB treatment showed an estimated IC50 of 76.56 mM; b) The fitted curve of NaP treatment showed an IC50 of 83.27 mM

## Slide 6
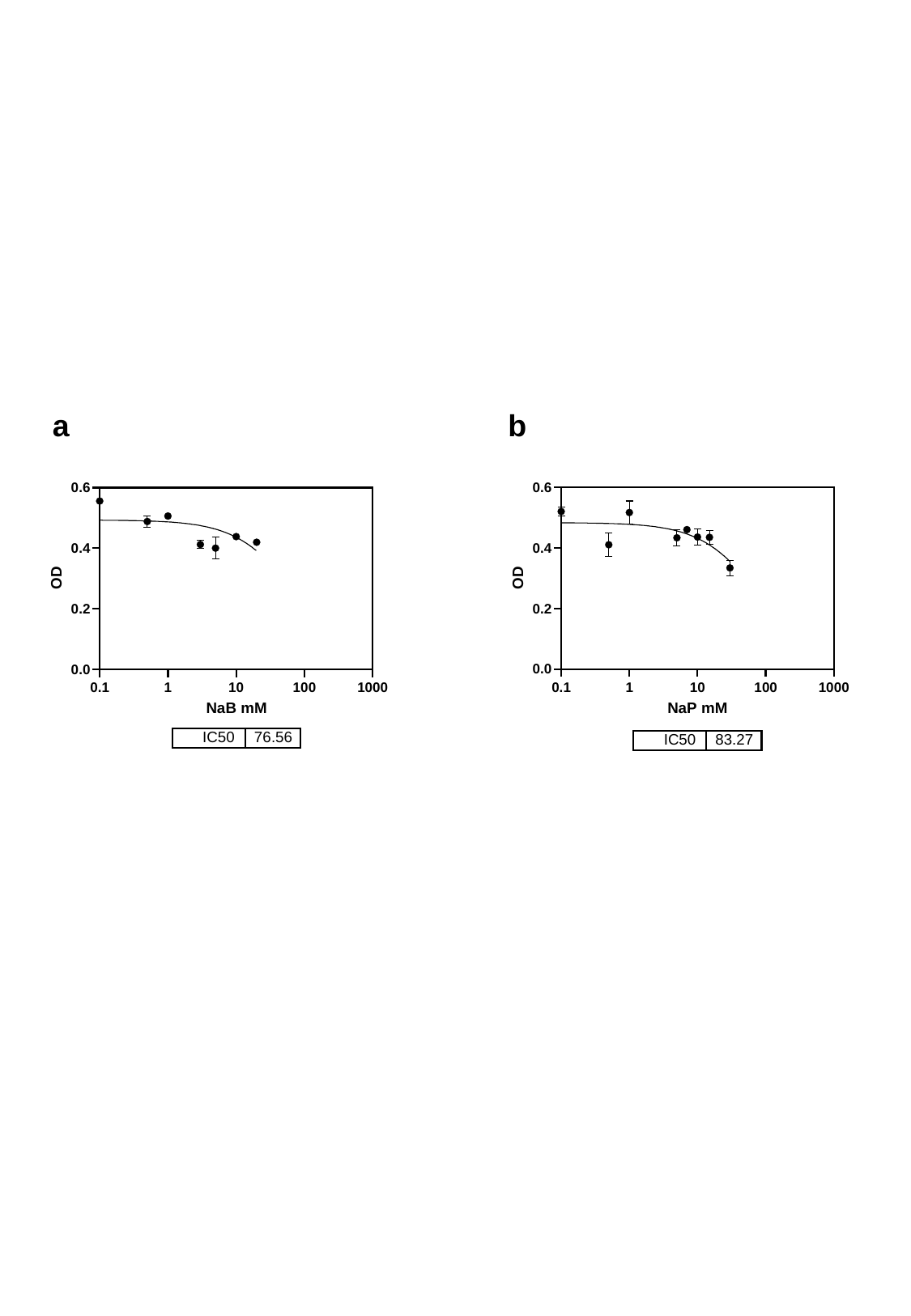

a
b
